# Comparison of the effectiveness of sacrospinous ligament fixation and sacrocolpopexy: a meta-analysis

**DOI:** 10.1007/s00192-021-04823-w

**Published:** 2021-06-03

**Authors:** Wenju Zhang, Willy Cecilia Cheon, Li Zhang, Xiaozhong Wang, Yuzhen Wei, Chaoxia Lyu

**Affiliations:** 1 Department of Gynecology and Obstetrics, The University of Hong Kong-Shenzhen Hospital, 1, Haiyuan 1st Road, Futian District, Shenzhen, 518053 Guangdong China; 2 Department of Gynecology and Obstetrics, Queen Elizabeth Hospital, 30 Gascoigne Road, Jordon, Kowloon, Hong Kong China

**Keywords:** Sacrocolpopexy, Sacrospinous ligament fixation, Meta-analysis

## Abstract

**Introduction and hypothesis:**

Sacrocolpopexy and sacrospinous ligament fixation (SSLF) have been used for the restoration of apical support. Studies comparing sacrocolpopexy and SSLF have reported conflicting results. We aim to assess the current evidence regarding efficiency and the complications of sacrocolpopexy compared with SSLF.

**Methods:**

We searched PubMed, Embase, and Cochrane Library and performed a systematic review meta-analysis to assess the two surgical approaches.

**Results:**

5Five randomized controlled trials, 8 retrospective studies, and 2 prospective studies including 4,120 cases were identified. Compared with abdominal sacrocolpopexy (ASC), SSLF was associated with a lower success rate (88.32% and 91.45%; OR 0.52; 95% CI 0.29–0.95; *p* = 0.03), higher recurrence (11.58% and 8.32%; OR 1.97; 95% CI 1.04–3.46; *p* = 0.04), and dyspareunia rate (14.36% and 4.67%; OR 3.10; 95% CI 1.28–7.50; *p* = 0.01). Patients in this group may benefit from shorter operative time (weighted mean difference −25.08 min; 95% CI −42.29 to −7.88; *p* = 0.004), lower hemorrhage rate (0.85% and 2.58%; OR 0.45; 95% CI 0.25–0.85; *p* = 0.009), wound infection rate (3.30% and 5.76%; OR 0.55; 95% CI 0.39–0.77; *p* = 0.0005), and fewer gastrointestinal complications (1.33% and 6.19%; OR 0.33; 95% CI 0.15–0.76; *p* = 0.009).

**Conclusion:**

Both sacrocolpopexy and SSLF offer an efficient alternative to the restoration of apical support. When anatomical durability and sexual function is a priority, ASC may be the preferred option. When considering factors of mesh erosion, operative time, gastrointestinal complications, hemorrhage, and wound infections, SSLF may be the better option.

## Introduction

Pelvic organ prolapse (POP) is a highly prevalent condition, which impairs the quality of life of patients significantly [1]. As a result of an aging population, the prevalence of women with POP will increase significantly, from 3.3 million to 4.9 million over the next 40 years [2]. Currently, more than 220,000 women seek surgical management for POP every year [3]. Prolapse may occur in the anterior vaginal wall (cystocele), posterior vaginal wall (rectocele), or at the apex (apical prolapse). Although cystocele appears to be the most frequent and recognized type of POP, the majority of women who suffer from cystocele at or beyond the hymen typically also have a component of apical support loss concomitantly [4, 5]. So the restoration of apical support is thought to be important for treating POP. There are several approaches to apical prolapse surgery, including abdominal sacrocolpopexy (ASC) and transvaginal sacrospinous ligament fixation (SSLF). Lane reported ASC in 1962 as an abdominal approach [6] and Richter described SSLF as a vaginal approach to apical prolapse in 1968 [7]. ASC is considered to be the gold standard treatment for apical prolapse. Numerous studies have shown that ASC had high success rates (78–100%) and long-term durability [8]. However, many surgeons choose to perform SSLF because of the shorter operative time and recovery [9]. Currently, laparoscopic sacrocolpopexy (LSC) develops rapidly to combine high success rates of ASC with better cosmetic satisfaction [10]. Despite several surgical approaches developed to restore apical support, there are no guidelines for which an apical support procedure should be performed and/or incorporated into a procedure designed to address prolapse. Currently, the choice of surgical approaches mostly depends on the preference and experiences of the surgeon. When discussing surgical options with patients, data comparing effectiveness and potential risks are important. Even though several studies comparing sacrocolpopexy (ASC and LSC) and SSLF have been reported, most are small series with conflicting results [6–11]. A comprehensive analysis that includes comparative data on both effectiveness and complications is still lacking. We performed a systematic review and meta-analysis on comparing sacrocolpopexy (ASC, LSC) and SSLF in women with apical prolapse.

## Materials and methods

### Literature search

The literature searches were last updated in October 2020 using MEDLINE, Embase, and the Cochrane Library. The following medical subject headings (MeSH) terms and their combinations were searched in [Title/Abstract]: “sacrospinous colpopexy,” “sacrospinous ligament fixation,” “sacrospinous ligament colpopexy,” “sacrospinous ligament suspension,” “sacrospinous hysteropexy,” “sacrospinous fixation,” and “sacrocolpopexy,” “colposacropexy,” “sacrohysteropexy,” and “sacral colpopexy.”

Supplementing the computer search, manual searches of the reference lists of all retrieved studies, review articles, and conference abstracts were performed. In the cases of repeated studies about the same population, the most recent or most informative report was used.

All comparative studies (randomized controlled trials [RCTs], case–control, or cohort studies) that compared sacrocolpopexy (ASC or LSC) with SSLF that had at least one of the outcomes mentioned in the next section of this paper, were included. Editorials, letters to the editor, review articles, case reports, meeting abstracts, and studies not published in the English language were excluded.

### Data extraction

For eligible articles, data were extracted and summarized independently by two reviewers (XZ Wang and YZ Wei). For each study, data were collected by one reviewer and a second reviewer confirmed the accuracy of the data. Any disagreement was resolved by the adjudicating senior authors (WJ Zhang and WC Cheon). After analyzing each study, the following data were collected:

1. Study characteristics
2. Patient characteristics
3. Intervention
4. Outcome definitions
5. Surgical outcomes and complications
6. Methodological quality items

### Quality assessment and statistical analysis

The methodological quality of the RCTs was determined by the Cochrane risk of bias tool [11]. The methodological quality of case–control and cohort studies was assessed by the modified Newcastle–Ottawa scale [12], which consists of three factors: method of patient selection, comparability of the study groups, and number of outcomes reported. A star rating of 0–9 was allocated to each study except for RCTs. RCTs and observational studies with seven or more stars were considered to be of high quality. Review manager 5.0 was used for meta-analyses. The odds ratio (OR) and weighted mean difference (WMD) were used to analyze dichotomous and continuous variables respectively.

We used 95% confidence intervals (CIs) for all outcomes. For dichotomous data, we used the numbers of events in the two groups to calculate Mantel–Haenszel odds ratios (ORs). For continuous data, we calculated the mean difference (MDs) and the standard deviations (STDs) using the technique described by Hozo et al. [13]. Statistical heterogeneity between studies was assessed using the Chi-squared test with significance set at *p* < 0.10, and heterogeneity was quantified using the I^2^ statistic. The random-effects model was reported if the *p* < 0.10. Otherwise, the fixed-effects model was reported. Subgroup analyses were performed to compare ASC and LSC with SSLF. Sensitivity analyses were performed for high-quality studies. Funnel plot analyses were used to determine the presence of publication bias.

## Results

**Fig. 1:**
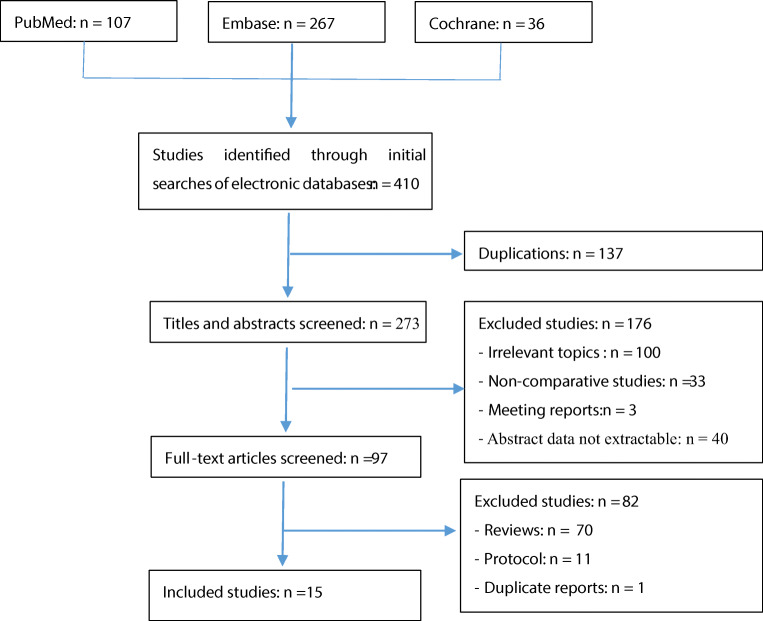
Flow diagram of studies identified, included, and excluded

Fifteen studies including 4,120 cases (2,409 cases for SSLF, 1,439 cases for ASC, and 272 cases for LSC) fulfilled the inclusion criteria and were included in the final analysis (Fig. 1) [14–28]. All publications were full-text articles. Examination of the references listed for these studies and for the review articles yields one study for evaluation [28]. Agreement between the two reviewers was 95% for study selection and 93% for quality assessment of trials.

The characteristics of the studies included are shown in Table 1. There were 5 RCTs [14, 20, 21, 25, 28] (level of evidence: 2b) [29]; 8 retrospective studies [15, 16, 18, 19, 22, 24, 26, 27] and 2 prospective studies (level of evidence: 2b–3b) [17, 23]. As for surgical procedures, 10 studies compared SSLF with ASC [15, 16, 18, 20–25, 28], 4 compared SSLF with LSC [14, 17,19, 26], and 1 compared ASC, LSC, and SSLF [27].

**Table 1:** Characteristics of the studies included

Study	Level of evidence	Design	Surgery	Patient number			Matching^a^	Follow-up, months	Quality score
				SSLF	ASC	LSC			
Benson and McClellan [29]	2b	RCT	SSLF/ASC	42	38		1,2,6,7	30	RCT
Biler et al. [27]	2b	R	SSLF/ASC/LSC	57	68	13	1,2,3,4,5,6 7	Perioperative	★★★★★★★
de Castro et al. [25]	1b	RCT	SSLF/ASC	35	36		1,2,3,4, 5,7	13.6	RCT
Chen et al. [26]	2b	R	SSLF/LSC	94		113	1,2,3,4, 6,7	24	★★★★★★★★
Demirci et al. [24]	3b	R	SSLF/ASC	60	45		1,2,3,4, 6, 7	Perioperative	★★★★★★
Eftekhar et al. [23]	3b	P	SSLF/ASC	39	23		1,2,4, 5,6	24	★★★★★★
van IJsselmuiden et al. [14]	1b	RCT	SSLF/LSC	58		59	1,2,3,4,5,6	12	RCT
Juliato et al. [22]	2b	R	SSLF/ASC	41	48		1,2,3,4, 5,6,7	6–9	★★★★★★★
Lo and Wang [21]	2b	RCT	SSLF/ASC	66	52		1,2,6,7	25	RCT
Maher et al. [20]	2b	RCT	SSLF/ASC	48	47		1,2,3,4,6,7	24	RCT
Marcickiewicz et al. [19]	3b	R	SSLF/ASC	51	60		1,2,3,4,6,7	36–60	★★★★★★★★
Ng and Han [18]	2b	R	SSLF/ASC	64	113		1,2,3,4,7	36	★★★★★★★
Ramanah et al. [17]	2b	P	SSLF/LSC	64		87	1,2,3,4, 6	30	★★★★★★
Sanses et al. [16]	3b	R	SSLF/ASC	1,642	863		1,4,5,6	12	★★★★★★
Sze et al. [15]	3b	R	SSLF/ASC	54	47		1,2,6,7	24	★★★★★★

*R* retrospective, *P* prospective study, *RCT* randomized controlled trial, *SSLF* sacrospinous ligament fixation, *ASC* abdominal sacrocolpopexy, *LSC* abdominal sacrocolpopexy

^a^Comparability variables: 1 = age; 2 = parity; 3 = body mass index; 4 = menopause; 5 = comorbidities; 6 = previous pelvic surgery history; 7 = concomitant surgical procedures

### Methodological quality of included studies

The quality of the studies included was variable. True randomization was used and other items in the risk of bias table are low risk in 5 RCTs [14, 20, 21, 25, 28]. However, none of the retrospective studies adopted an appropriate protocol for treatment assignment, with allocation usually at the discretion of the surgeon. Matching criteria between the groups were variable, which may have introduced selective bias. All studies mentioned the length of follow-up. However, 2 of the studies provided only perioperative data [24, 27] and 4 studies provided a short follow-up, which is less than 2 years [14, 16, 22, 25]. Methods for handling missing data were not adequately described in some studies [15, 17, 23, 24].

The outcomes of meta-analysis comparison of SSLF and sacrocolpopexy are shown in Table 2.

**Table 2:** Results of meta-analysis comparison of sacrospinous ligament fixation (SSLF) and sacrocolpopexy

Outcomes of interest	Study, number	SSLF patients, number	Sacrocolpopexy patients, number	WMD/OR (95% CI)	*p* value^*^	Study heterogeneity			
						Chi-squared	df	I^2^, %	*p* value
OT, min	8	515	540	−31.67 (−48.69, −14.65)	**<0.00003**	125.33	8	94	**<0.00001**
Hemorrhage	5	312	340	0.46 (0.19, 1.10)	0.08	3.97	5	0	0.55
Dyspareunia	6	266	171	2.26 (1.19, 4.30)	**0.01**	9.38	6	36	0.15
Gastrointestinal complications	5	331	290	0.59 (0.28, 1.22)	0.16	2.01	4	0	0.73
Wound infection	6	391	429	0.46 (0.21, 1.02)	0.06	5.59	5	11	0.35
Tissue injury	6	301	345	1.45 (0.65, 3.25)	0.37	3.24	5	0	0.66
Recurrence	8	521	550	2.26 (1.10, 4.65)	**0.03**	13	6	54	**0.04**
Success	8	521	550	0.47 (0.25, 0.89)	**0.02**	11.27	6	47	0.08

*OT* operative time, *WMD/OR* weighted mean difference/odds ratio, *df* degrees of freedom, *CI* confidence interval

*Statistically significant results are shown in bold

Ten studies including 1,132 patients reported operative time [15, 18–21, 24–28]. The operation time was significantly shorter in the SSLF group than in the ASC group (WMD −25.08 min; 95% CI −42.29 to −7.88; *p* = 0.004). Four studies assessed operation time in 419 patients show no significant difference between SSLF and LSC (WMD: −37.56 min; 95% CI, −81.04 to 5.93; *p* = 0.09).

**Fig. 2:**
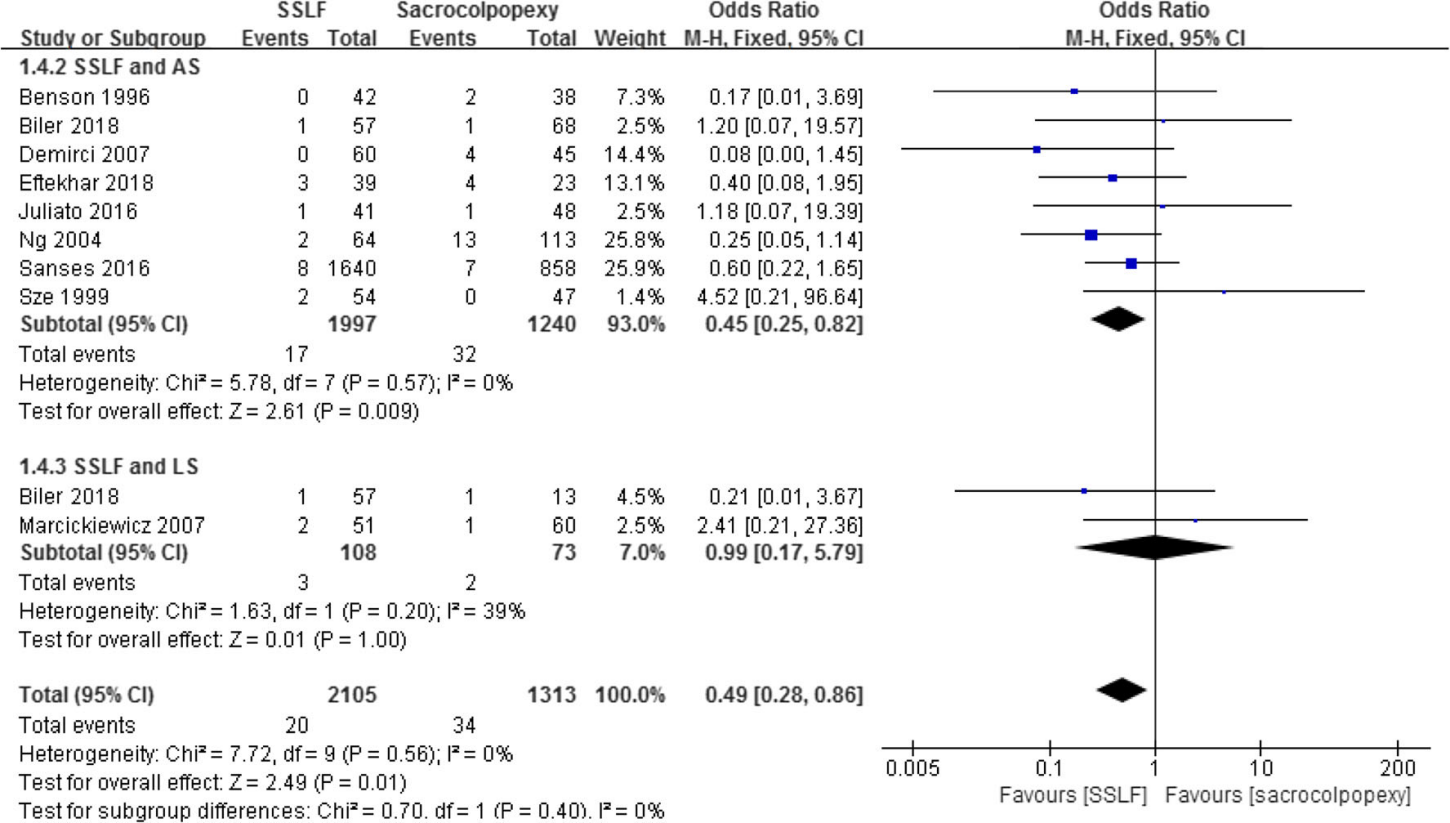
Forest plot and meta-analysis of hemorrhage rates. *SSLF* sacrospinous ligament fixation, *AS* abdominal sacrocolpopexy, *LS* laparoscopic sacrocolpopexy, *M–H* Mantel–Haenszel method, *CI* confidence interval

Nine studies assessed hemorrhage in 3,418 patients showed that there was a significant difference between the SSLF and sacrocolpopexy groups (0.95% and 2.59%; OR 0.49; 95% CI 0.28–0.86; *p* = 0.01) [15, 16, 18, 19, 22–24, 27, 28]. When patients were divided into SSLF/ASC and SSLF/LSC subgroups, there was a significant difference between SSLF and ASC (0.85% and 2.58%; OR 0.45; 95% CI 0.25–0.85; *p* = 0.009) [15, 16, 18, 22–24, 27, 28,], but no difference between SSLF and LSC (2.78% and 2.74%; OR 0.99; 95% CI 0.17–5.79; *p* = 1.0; Fig.2) [19, 27].

**Fig. 3:**
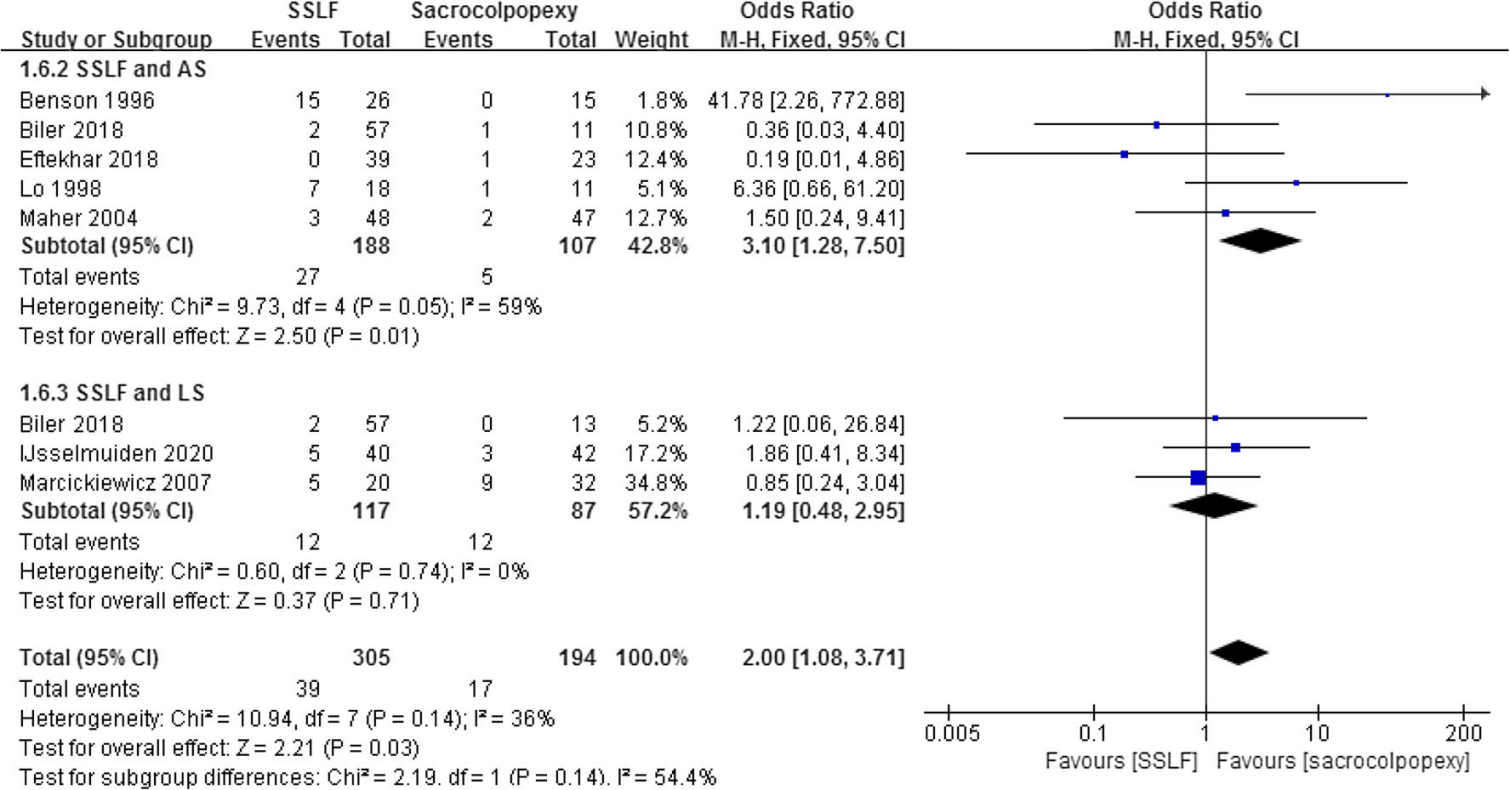
Forest plot and meta-analysis of dyspareunia rates. *SSLF* sacrospinous ligament fixation, *AS* abdominal sacrocolpopexy, *LS* laparoscopic sacrocolpopexy, *M–H* Mantel–Haenszel method, *CI* confidence interval

Seven studies including 499 patients reported dyspareunia [14, 19–21, 23, 27, 28]. There was a significant difference between SSLF and sacrocolpopexy groups (12.79% and 8.76%; OR 2.00; 95% CI 1.08–3.71; *p* = 0.03). When patients were divided into SSLF/ASC and SSLF/LSC subgroups, there was a significant difference between SSLF and ASC (14.36% and 4.67%; OR 3.10; 95% CI 1.28–7.50; *p* = 0.01), but no difference between SSLF and LSC (10.26% and 13.79%; OR 1.19; 95% CI 0.48–2.95; Fig. 3).

**Fig. 4:**
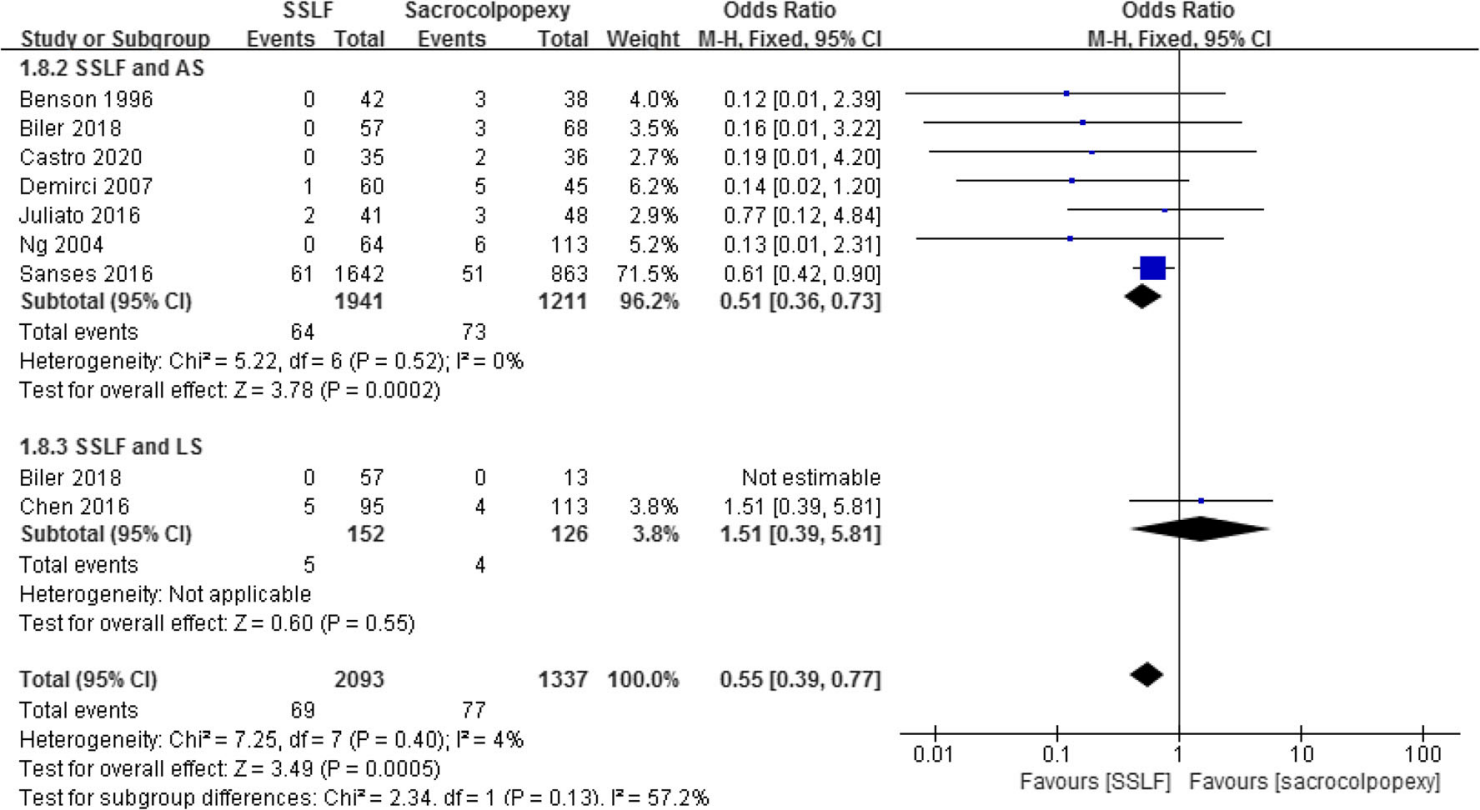
Forest plot and meta-analysis of wound infection rates. *SSLF* sacrospinous ligament fixation, *AS* abdominal sacrocolpopexy, *LS* laparoscopic sacrocolpopexy, *M–H* Mantel–Haenszel method, *CI* confidence interval

Eight studies including 3,430 patients reported wound infection [16, 18, 22, 24–28]. There was a significant difference between the SSLF and sacrocolpopexy groups (3.30% and 5.76%; OR 0.55; 95% CI 0.39–0.77; *p* = 0.0005; Fig. 4). Wound infection rates were available in seven studies in the subgroup of SSLF and ASC, which also showed a significant difference (3.30% and 6.03%; OR 0.51; 95% CI 0.36–0.73; *p* = 0.0002). However, the data from the SSLF/LSC subgroup showed no significant difference in wound infection rates between SSLF and LSC (3.29% and 3.17%; OR 1.51; 95% CI 0.39–5.81; *p* = 0.55; Fig. 4).

Gastrointestinal complications included the symptoms of ileus, a bowel obstruction after the operation. Seven studies including 3,220 patients reported gastrointestinal complications [14–16, 19, 21, 27, 28]. The difference in gastrointestinal complications was significantly lower in SSLF than in ASC (1.33% and 6.19%; OR 0.33; 95% CI 0.15–0.76; *p* = 0.009).

Tissue injury included bladder, ureter, and bowel injuries during the operation. Nine studies including 3,318 patients reported tissue injuries [16, 18–21, 23–25, 28]. There was no difference between the SSLF and sacrocolpopexy groups (4.95% and 5.25%; OR 0.87; 95% CI 0.63–1.19; *p* = 0.38). Eight studies comparing SSLF with ASC reported tissue injuries. There was no difference (5.02% and 5.35%; OR 0.87; 95% CI 0.63–1.20; *p* = 0.41). Only one study comparing SSLF and LSC reported tissue injury and there was no difference between SSLF and LSC.

Pooling the data from 12 studies that assessed recurrence in 3,890 patients showed that the recurrence rate in SSLF was significant higher than in the sacrocolpopexy group (11.34% and 7.90%; OR 1.96; 95% CI 1.10–3.47; *p* = 0.02) [14–18, 20–23, 26–28]. When the sacrocolpopexy group was divided into ASC and LSC subgroups, the difference in the recurrence rate was statistically significant in favor of ASC when compared with SSLF (11.58% and 8.32%; OR 1.97; 95% CI 1.04–3.46; *p* = 0.04), with no significant difference between SSLF and LSC (9.52% and 5.88%; OR 2.03; 95% CI 0.37–11.19; *p* = 0.42).

**Fig. 5:**
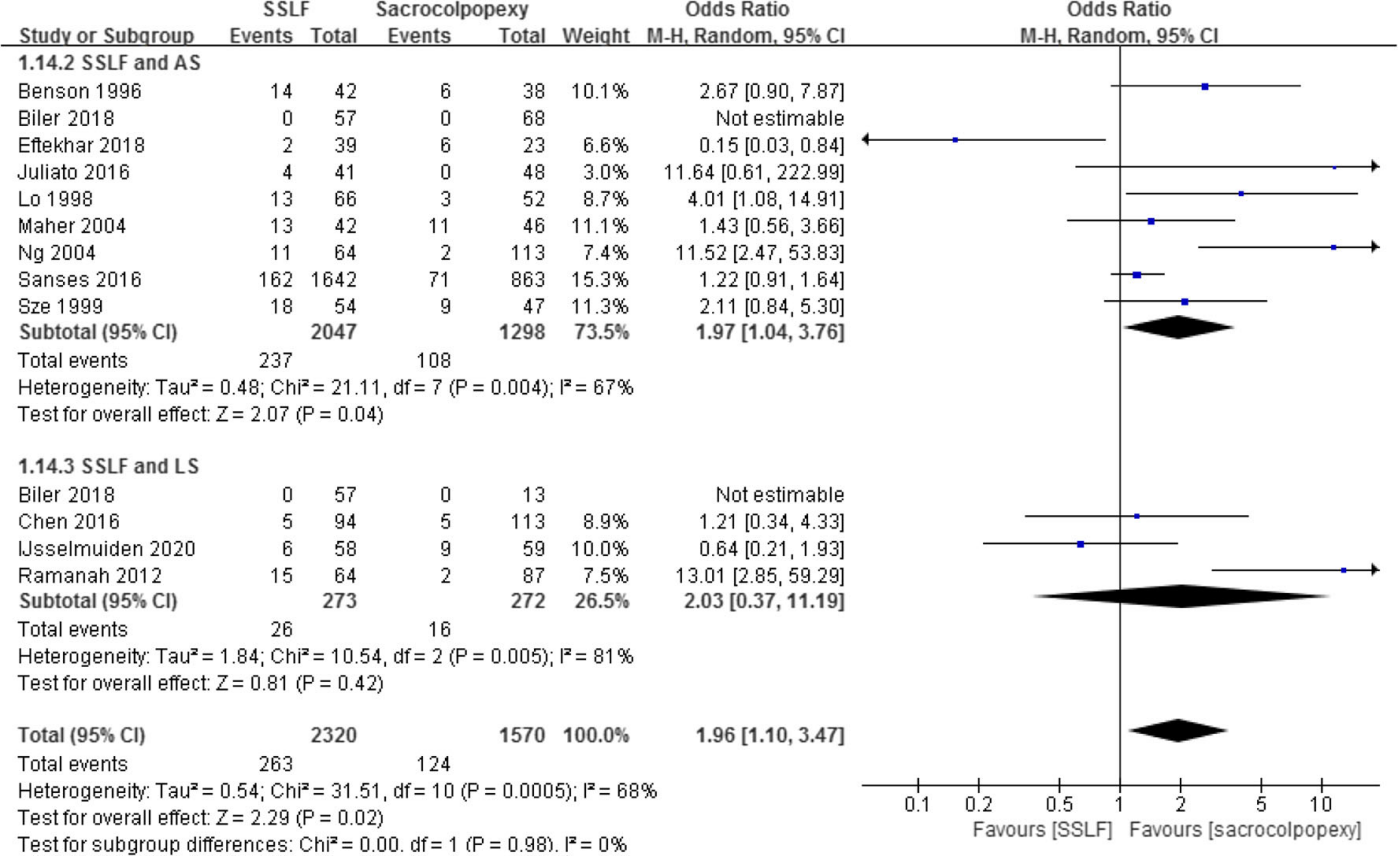
Forest plot and meta-analysis of vault prolapse recurrence rates. *SSLF* sacrospinous ligament fixation, *AS* abdominal sacrocolpopexy, *LS* laparoscopic sacrocolpopexy, *M–H* Mantel–Haenszel method, *CI* confidence interval

The pelvic organ prolapse recurrences were divided into vault prolapse, cystocele, and rectocele recurrence in five studies, including 491 patients [14, 19, 20, 22, 25]. Three studies including 255 patients reported on vault prolapse recurrences in the SSLF and ASC groups [20, 22, 25]. The difference was statistically significant in favor of ASC (OR 3.31 95% CI 1.04–10.50; *p* = 0.04; Fig. 5). The difference was no significant difference in cystocele recurrence (OR 1.65; 95% CI 0.83–3.28; *p* = 0.15) and rectocele recurrence (OR 0.60; 95% CI 0.06–5.63; *p* = 0.66) between SSLF and ASC.

Two studies including 236 patients reported on vault prolapse, cystocele, and rectocele recurrences in SSLF and LSC. There was no significant difference in the vault prolapse recurrence(OR 3.20; 95% CI 0.13–80.03; *p* = 0.48; Fig. 5), cystocele recurrence (OR 0.94; 95% CI 0.40–2.19; *p* = 0.88), and rectocele recurrence (OR 0.12; 95% CI 0.01–2.32; *p* = 0.16) between SSLF and LSC.

**Fig. 6:**
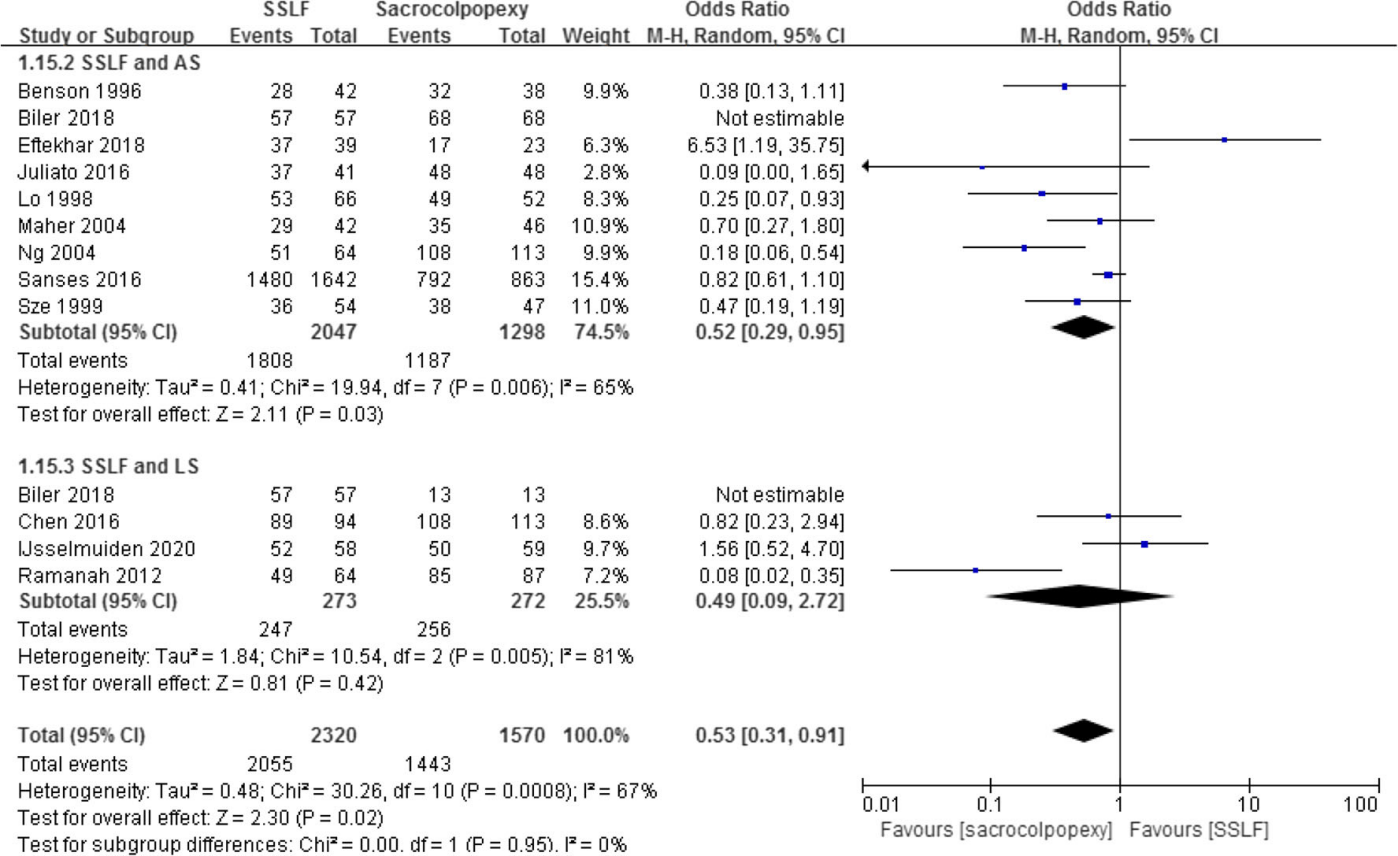
Forest plot and meta-analysis of success rates. *SSLF* sacrospinous ligament fixation, *AS* abdominal sacrocolpopexy, *LS* laparoscopic sacrocolpopexy, *M–H* Mantel–Haenszel method, *CI* confidence interval

Pooling the data from 12 studies that assessed success rates in 3,890 patients showed that the success rates in SSLF were significantly lower than in the sacrocolpopexy group (88.58% and 91.91%; OR 0.53; 95% CI 0.31–0.91; *p* = 0.02) [14–18, 20–23, 26–28]. When the sacrocolpopexy group was divided into the ASC and LSC subgroups, there was still a significant difference between SSLF and ASC (88.32% and 91.45%; OR 0.52, 95% CI 0.29–0.95; *p* = 0.03), but no difference between SSLF and LSC (90.48% and 94.12%; OR 0.49; 95% CI 0.09–2.72; *p* = 0.42; Fig. 6).

Five RCTs [14, 20, 21, 25, 28] and five retrospective studies [18, 19, 22, 26, 27] that scored seven or more stars on the modified Newcastle–Ottawa scale were involved in a sensitivity analysis (Table 3). There was no change in the significance of the outcomes except for hemorrhage rate, wound infection rate, and gastrointestinal complications. There was no significant difference in hemorrhage rate (OR 0.46; 95% CI 0.19–1.10; *p* = 0.08), wound infection rate (OR 0.46; 95% CI 0.21–1.02; *p* = 0.06) and gastrointestinal complications (OR 0.59; 95% CI 0.28–1.22; *p* = 0.16) between SSLF and sacrocolpopexy in the sensitivity analysis.

**Table 3:** Sensitivity analysis comparison of sacrospinous ligament fixation (SSLF) and sacrocolpopexy

Outcomes of interest	Study, number.	SSLF, patient, number	Sacrocolpopexy, patients, number	WMD/OR (95% CI)	*p* value*	Study heterogeneity			
						χ2	df	I^2^, %	*p* value
OT, min	8	515	540	−31.67 (−48.69, −14.65)	**<0.00003**	125.33	8	94	<0.00001
Hemorrhage	5	312	340	0.46 (0.19, 1.10)	0.08	3.97	5	0	0.55
Dyspareunia	6	266	171	2.26 (1.19, 4.30)	**0.01**	9.38	6	36	0.15
Gastrointestinal complications	5	331	290	0.59 (0.28, 1.22)	0.16	2.01	4	0	0.73
Wound infection	6	391	429	0.46(0.21, 1.02)	0.06	5.59	5	11	0.35
Tissue injury	6	301	345	1.45 (0.65, 3.25)	0.37	3.24	5	0	0.66
Recurrence	8	521	550	2.26 (1.10, 4.65)	**0.03**	13	6	54	0.04
Success	8	521	550	0.47(0.25, 0.89)	**0.02**	11.27	6	47	0.08

*OT* operative time, *WMD/OR* weighted mean difference/odds ratio, *df* degrees of freedom, *CI* confidence interval

*Statistically significant results are shown in bold

The degree of between-study heterogeneity decreased significantly for gastrointestinal complications and success rate. Between-study heterogeneity showed no change for operative time, hemorrhage, dyspareunia, tissue injury, and recurrence rates.

**Fig. 7:**
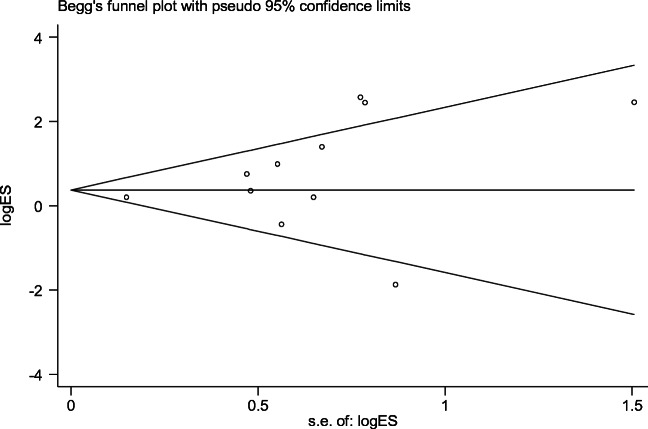
Funnel plot illustrating a meta-analysis of the recurrence rates. *s.e.*. standard error

Figure 7 showed a funnel plot of the studies included in this meta-analysis that reported recurrence rates. The funnel plot was drawn and the *p* value of bias was calculated using Stata/SE software. In the funnel plot, most studies lie inside the 95% CIs, with an even distribution around the horizontal, and *p* value = 0.202. Both indicate no obvious publication bias.

## Discussion

This systematic review and meta-analysis shows that ASC has better anatomical results and lower recurrence than does SSLF. Furthermore, the recurrence of apical prolapse, cystocele, and rectocele were assessed in ASC and SSLF separately. There were no significant differences between the groups in cystocele or rectocele recurrence; however, there was a significantly increased recurrence and lower success rate of apical prolapse for SSLF compared with ASC. The results demonstrate that ASC offers greater support to the apex than SSLF. Sacrocolpopexy suspends the apex with mesh secured to the anterior longitudinal sacral ligament or at the sacral bone (S2), the greater support to the apex that is afforded by the strong mesh may account for the lower rate of both apical and overall prolapse recurrence and higher success rate in the sacrocolpopexy compared with SSLF.

A second factor for consideration may be the possible effect of neuropathy produced by massive vaginal dissection in SSLF. The findings of neuropathy with vaginal dissection have been reported [29, 30], such neuropathy may have a negative effect on pelvic muscle, fascia, and ligament. Attenuation and subsequent weakening of the pelvic support due to neuropathy, aging, menopause, or inherent weakness of the supporting tissue, may cause the recurrence of SSLF.

There were no differences between LSC and SSLF in apical prolapse, cystocele, rectocele, overall recurrence, or success rate. Laparoscopic sacrocolpopexy is a minimally invasive surgery that is an innovative and developing technique. It involves a high level of expertise and is associated with longer learning curves. It is also more challenging for surgeons than SSLF or abdominal sacrocolpopexy.

The following two factors may contribute to the difference in LSC and ASC results: first, insufficient pulling of the mesh; second, mesh displacement. With regard to the first factor, in a special surgical environment under laparoscopy, it is often difficult to pull the mesh onto the sacral promontory with good strength. This could cause loosening of the mesh and lead to recurrence. Mesh displacement can occur as a result of weak mesh fixation on the vaginal cuff or cervical stump or a loss of tension within the absorbable thread, which is more likely to occur in LSC than in ASC. Moreover, the small number of studies (only 4) involved in the analysis may contribute to bias, which requires further studies to confirm the conclusion.

Both of SSLF and LSC are minimally invasive surgery with the avoidance of a large abdominal wound, resulting a in better cosmetic outcome. There are no significant differences in adverse event rates between the groups, except that LSC has a lower febrile rate than SSLF. This finding indicates that LSC is at least as safe and efficient as SSLF. However, when starting LSC, proper patient selection, adequate laparoscopic experience, and preferably a certain amount of LSC training are recommended to minimize recurrence and improve the success rates.

The dyspareunia rate was significantly higher in SSLF than in ASC. Excessive vaginal dissection, concurrent with overzealous repairs of cystocele, rectocele, or perineoplasty in SSLF may result in neuropathy and extensive vaginal scarring. Owing to scar contraction, the vagina becomes shorter and narrower. These factors result in more dyspareunia in SSLF than in ASC.

Despite all these clinical benefit, ASC involves a longer operative time and more hemorrhage, wound infection, and gastrointestinal complications than SSLF. Furthermore, it is associated with synthetic mesh erosion [31, 32] and higher costs.

To assess the impact of study quality on the effect estimates, we performed a sensitivity analysis including only high-quality studies. Most of the results were similar to those of the analysis. Although a meta-analysis of RCTs only would be ideal, the limited number of RCTs prevented us from reaching any definitive conclusions. Between-study heterogeneity was not significant for hemorrhage, dyspareunia, gastrointestinal complications, wound infection, and issue injury, but was significant for success and recurrence. Different definitions of success and recurrence were adopted in the studies included, which might contribute to the significant between-study heterogeneity. Pooling of data using the random-effects model might reduce the effect of heterogeneity but cannot abolish it completely.

We acknowledge some limitations. First, the primary outcomes of success and recurrence were defined by each study, and most trials reported different definitions of success and recurrence. These definitions of success included no prolapse greater or equal to grade 2 at any vaginal site [20], the achievement of POP stage 0 or 1 [26], asymptomatic with equal or less than grade 1 vault prolapse based on the halfway system [18], less than or equal to stage 2 [22], no protrusion of the vaginal wall greater than that in stage II according to the ICS grading system [21], no prolapse beyond the hymen, no bothersome bulge symptoms, and no therapy for recurrent prolapse within 12 months [14], freedom from symptoms, vaginal apex remained above the levator plate with no protrusion of any vaginal tissue beyond the hymen [28], and grade 1 or no prolapse of the vault at the time of follow-up based on a modified version of the Baden–Walker system [19]. It is known that treatment success and recurrence varies widely depending on the definition. Variation in success and recurrence definitions make it difficult to make comparisons.

Second, the number of women with only ASC/LSC or SSLF was small. Most patients have various other procedures as well, including abdominal total hysterectomy, total vaginal hysterectomy, anterior colporrhaphy, posterior colporrhaphy, Burch colposuspension, tension-free vaginal tape, paravaginal cystocele repair, etc. These concomitant procedures introduce an outcome bias and may negatively affect the accuracy of different outcomes, such as success and recurrence rate, operative time, dyspareunia, etc.

Third, most of the studies involved were retrospective, except for five RCTs. Inadequate random sequence generation and blinding tended to increase the risk of bias, which may negatively affect the accuracy of the results.

Finally, studies published in non-English language were excluded, which may lead to a possible publication bias. To verify this, a funnel plot is drawn and the *p* value of bias is calculated in Stata/SE software. Neither shows any significant publication bias.

The strengths are as follows. First, most of the studies provided an adequate follow-up period. The adequate follow-up period for outcome of interest can decrease the risk of bias. Studies have shown that the success rate gradually declines with time, but almost 95% of recurrences occur within 2 years [33]. In our studies, 11 of the studies included have a follow-up period of more than 1 year and 9 more than 2 years, which increased the accuracy of the outcomes.

Second, our study includes all studies published in English comparing SSLF and sacrocolpopexy in this area, with enough data accumulated for inspection. The most comprehensive and up-to-date information decreases the risk of bias on the assessment of surgical efficacy.

Finally, we performed a comprehensive assessment of adverse events, which is helpful as comparative studies are often underpowered to assess infrequent adverse events. The strength of this approach is exemplified by our results of “hemorrhage.” In most studies, it appears that there are higher odds of hemorrhage after sacrocolpopexy; however, there is no significant difference between groups in any studies owing to rare cases of hemorrhage in each study. When including all studies that reported hemorrhage, which essentially increases the sample size, the difference was statistically significant in favor of SSLF.

## Conclusion

Based on this meta-analysis, when anatomical durability and sexual function are priorities, ASC may be the preferred option for surgical reconstruction of apical prolapse. When considering the factors of mesh erosion, the cost of mesh, operative time, hemorrhage, wound infection, gastrointestinal complications, and better cosmetic satisfaction, SSLF may be the better option. Further studies on LSC are awaited to confirm its efficacy and adverse events.
